# Estimation of the Attributable Fraction of Noncommunicable Diseases Related to Physical Inactivity, Morocco

**DOI:** 10.5888/pcd23.250352

**Published:** 2026-05-14

**Authors:** Nassiba Bahra, Fatima Zahrae Bartal, Hajar Mahfoudi, Karima El Rhazi, Nabil Tachfouti

**Affiliations:** 1 Laboratory of Epidemiology, Clinical Research, and Community Health, Faculty of Medicine, Pharmacy, and Dentistry, Sidi Mohamed Ben Abdellah University, Fez, Morocco; 2 Diagnostic Center, Hassan II University Hospital, Fez, Morocco; 3 Laboratory of Epidemiology and Health Sciences Research, Sidi Mohamed Ben Abdellah University, Fez, Morocco

## Abstract

**Introduction:**

Physical inactivity is a major risk factor for noncommunicable diseases (NCDs). With the growing prevalence of NCDs, quantifying the effects of physical inactivity is essential for informing public health policies. This study estimates the attributable fraction of NCDs related to physical inactivity in Morocco and examines differences by sex and physical activity level.

**Methods:**

We applied a population attributable fraction approach to estimate preventable cases of breast cancer, colorectal cancer, type 2 diabetes, ischemic heart disease, and ischemic stroke through increased physical activity. The analysis focused on adults aged 18 years or older, with a focus on ischemic conditions among those aged 35 or older. Data sources included the national World Health Organization (WHO) STEPwise approach to NCD risk factor surveillance (STEPS) survey (for inactivity prevalence), meta-analyses (for relative risks), the national cancer registry (for cancer incidence), and published studies (for disease prevalence).

**Results:**

For breast cancer in women, physical inactivity accounted for 0.88% to 3.95% of cases. For colorectal cancer, the attributable fraction ranged from 2.72% (women) and 1.69% (men) at low activity levels to 6.49% (women) and 4.13% (men) at high activity levels. For type 2 diabetes, it ranged from 4.16% (women) and 2.60% (men) to 9.10% (women) and 5.84% (men). Among adults aged 35 years or older, physical inactivity accounted for up to 6.49% of ischemic heart disease cases and 7.08% of ischemic stroke cases in women and up to 5.16% and 5.64%, respectively, in men.

**Conclusions:**

Higher physical activity levels could significantly reduce NCD incidence. These findings underscore the preventive potential of physical activity and the need for sex-sensitive public health strategies to reduce NCD burden.

**Summary**

**What is already known about this topic?**

Physical inactivity is a major risk factor for noncommunicable diseases worldwide and contributes substantially to the global burden of disease. However, limited evidence of risks is available from low-income and middle-income countries, including Morocco.

**What is added by this report?**

This study estimates, for the first time in Morocco, the attributable fraction of major noncommunicable diseases related to physical inactivity, providing national-level evidence on preventable disease burden.

**What are the implications for public health practice?**

Our findings highlight the urgent need to strengthen policies and interventions promoting physical activity in Morocco to reduce the burden of chronic diseases and improve population health outcomes.

## Introduction

Physical inactivity is a major public health issue affecting a substantial proportion of the global population. According to the World Health Organization (WHO), physical inactivity is the fourth leading risk factor for noncommunicable diseases (NCDs) and is responsible for 3.2 million to 5 million deaths annually (1). Approximately 31% of the global population does not meet the recommended levels of physical activity needed to benefit from its health-protective effects (1). According to France’s National Agency for Food, Environmental and Occupational Health and Safety, 95% of adults in France are exposed to health risks from insufficient physical activity or prolonged sedentary behavior (2).

The health effects of insufficient physical activity are substantial. Physical inactivity is associated with an increased risk of several chronic conditions, including cardiovascular disease, cancer, and type 2 diabetes (3,4) Prolonged sedentary behavior is also associated with an increased risk of all-cause mortality, particularly at higher levels of daily sitting time (5). 

Physical inactivity is now recognized as a global public health challenge requiring urgent action (6). Worldwide, physical inactivity is responsible for approximately 27% of diabetes cases, 30% of ischemic heart disease cases, and 21% to 25% of breast and colon cancer cases (4). In addition, physically inactive people have a 20% to 30% higher risk of all-cause mortality than physically active people (4). In response, WHO established a global target of a 10% relative reduction in insufficient physical activity by 2025 (6).

The importance of physical activity in the management of NCDs has been well documented. Exercise is known to enhance physiological well-being as well as cognitive, emotional, social, and psychosocial health. In addition, it can shift perceptions about health and improve acceptance and coping with chronic illnesses (7–9).

NCDs are responsible for two-thirds of global deaths. Approximately 80% of NCD-related deaths — totaling around 30 million annually — occur in low-income and middle-income countries. Several factors contribute to the development of NCDs, including diet, lifestyle, and physical inactivity, which alone accounts for 9% of all deaths worldwide (4).

In Morocco, the situation is no different. The country is undergoing an epidemiologic transition, marked by a rising prevalence of NCDs, which now account for most illness and death. The WHO STEPwise approach to NCD risk factor surveillance (STEPS), conducted by the Ministry of Health, indicated that physical inactivity is one of the leading contributors to this growing prevalence of NCDs (10). This trend is largely driven by modifiable behavioral risk factors, such as smoking, physical inactivity, and unhealthy diets. These factors pose a significant challenge to the public health system and play a critical role in the rise of chronic diseases, making their prevention and management a public health priority (10).

Calculating the attributable fraction and the number of disease cases linked to physical inactivity is crucial for assessing its impact on public health. The attributable fraction measures the proportion of deaths and illnesses that could be prevented by reducing physical inactivity in a given population. This metric is essential for understanding the causal relationship between physical inactivity and the development of various pathologies, such as cardiovascular diseases, diabetes, obesity, and certain types of cancer. Furthermore, estimating the number of illnesses attributable to physical inactivity provides a concrete assessment of the health burden imposed by this risk factor (11–13).

Understanding the role of physical inactivity in the development of NCDs enables the design of more effective primary prevention strategies, maximizes public health benefits, and ensures the optimal allocation of available resources. In this study, we estimated the attributable fraction of NCDs linked to physical inactivity in Morocco and examined differences by sex and level of physical activity.

## Methods

We first identified the major diseases most strongly associated with physical inactivity on the basis of evidence from WHO and existing literature. These are breast cancer, colorectal cancer, type 2 diabetes, ischemic heart disease, and ischemic stroke (14).

According to WHO, physical inactivity is defined as not achieving the recommended level of physical activity for health (15). WHO recommends a minimum of 600 metabolic equivalent minutes (MET-min/wk) of total activity per week to achieve health benefits. This is equivalent to at least 150 minutes per week of moderate-intensity activity, 75 minutes per week of rigorous-intensity activity, or an equivalent combination (15).

We used a population-based approach to estimate the health burden of physical inactivity by quantifying the proportion of cases of these diseases that could be prevented through increased physical activity. This estimation was based on the concept of the population attributable fraction (PAF), an epidemiologic measure that is used to assess the effect of a risk factor on the incidence or prevalence of a disease in a given population. The PAF represents the proportion of cases that could be prevented if the specific risk factor were eliminated. By providing quantitative estimates, the PAF enables policymakers to evaluate the potential effectiveness of interventions aimed at reducing or eliminating the risk factor (16). The analysis integrated multiple data sources, including national survey data on physical inactivity, relative risks (RRs) from meta-analyses, and national incidence and prevalence data for the studied diseases.

This study was based on secondary data analysis using aggregated, nonidentifiable national statistics. Therefore, approval from an institutional review board was not required.

### Modeling

We adopted a population-based approach to assess the health effects of physical inactivity. To this end, we calculated a semi-adjusted PAF for each outcome by using a method that combines the crude PAF formula with a multivariable-adjusted RR, an approach reported to have low relative bias (17). Specifically, the PAF was determined by using the formula

PAF = P × (adjusted RR − 1) **/** [1 + P × (adjusted RR − 1)]

where P represents the prevalence of physical inactivity in the study population and RR is the multivariable-adjusted RR. We considered 2 RRs corresponding to 2 levels of physical activity to assess the differential effect of physical inactivity: 1) comparison between physical inactivity and a low level of physical activity, and 2) comparison between physical inactivity and a high level of physical activity.

Illness attributable to physical inactivity is expressed as the number of disease cases linked to physical inactivity. The number of cases attributable to physical inactivity (N_attributable_) was calculated for each disease by using the formula

N_attributable_ = N_disease_ × PAF

where N_disease_ represents the total number of disease cases and PAF is the population attributable fraction.

### Data source

Data on the prevalence of physical inactivity were obtained from the STEPS survey (10). Physical activity was assessed by using the Global Physical Activity Questionnaire (GPAQ), the standardized instrument used within the STEPS survey to measure physical activity, and the definition of physical inactivity aligns with WHO recommendations: less than 150 minutes of moderate-intensity physical activity or 75 minutes of vigorous-intensity physical activity per week, or an equivalent combination of both.

The STEPS survey is a large-scale national study conducted in Morocco in 2017. It was carried out on a representative sample of the Moroccan population. The primary objective of the survey was to assess several risk factors for NCDs, such as obesity, smoking, sedentary behavior, and fruit and vegetable consumption. The data collected during the survey provided valuable insights into the health status and health-related behaviors of the Moroccan population (10).

### Relative risks

We obtained summary RR estimates, adjusted for the effects of physical inactivity on each outcome, from a study by Kyu et al (18). This study was a systematic review and dose–response meta-analysis to quantify the association between physical activity and the risk of breast cancer, colon cancer, diabetes, ischemic heart disease, and ischemic stroke, as part of the Global Burden of Disease Study 2013. The authors used a definition of physical inactivity that aligns with WHO recommendations (<600 MET-min/wk or <150 min/wk) and included 174 prospective cohort studies from 1980 through 2016 (18).

This meta-analysis presented RRs for each level of physical activity, demonstrating a protective effect of physical activity. We used the RRs corresponding to a low level of physical activity (600–3,999 MET-min/wk) and a high level of physical activity (≥8,000 MET-min/wk). To assess the impact of physical inactivity, we inverted these RRs, thereby obtaining the RRs associated with physical inactivity compared with the reference levels of high and low physical activity. This method is frequently used to evaluate the RRs associated with physical inactivity in comparison to different levels of physical activity (4).

### Morbidity data

The number of new cases of breast cancer and colorectal cancer was calculated by multiplying the standardized incidence rates for the Moroccan population, as reported in the Greater Casablanca Cancer Registry (2018–2021) (19), by the total population size in 2018, according to data from the High Commission for Planning (20).

The number of prevalent cases of type 2 diabetes was estimated by using the prevalence of diabetes reported in a study conducted to assess the prevalence and associated factors of type 2 diabetes among adults in Morocco (21). The study analyzed cross-sectional data from a representative sample of 4,779 people aged 18 years or older who participated in the national STEPS survey in Morocco in 2017 and had completed fasting blood glucose measurement (21).

The prevalent number of cases of ischemic heart disease was obtained by using data from a national study on the epidemiology of cardiovascular disease conducted during 2000 through 2016, supplemented by data from the international cardiovascular disease burden study (22,23).

The number of cases of ischemic stroke was estimated based on the standardized prevalence of stroke in the Moroccan population aged 35 years or older (24).

### Sensitivity analysis

A univariate sensitivity analysis was conducted to assess the robustness of the results by varying the values of 2 key parameters: sex and level of physical activity. Sex was included as a parameter because the prevalence of physical inactivity varies significantly between men and women (25). The level of physical activity was also examined, as low and high levels of activity have distinct effects on the rates of NCDs. The results of this analysis were visualized by using a tornado diagram, which illustrates the impact of variations in these parameters on the attributable fraction. This approach allowed us to evaluate how changes in these parameters influence estimates of preventable disease cases, thereby ensuring the reliability and relevance of our conclusions.

## Results

### Physical inactivity

According to the 2017 STEPS survey, the prevalence of physical inactivity among Moroccan adults aged 18 years or older was higher among women, at 26.0%, than among men, at 16.1%. For the calculation of the PAF for ischemic heart disease and ischemic stroke, prevalence estimates were recalculated for an older age stratum to align with the available case data for adults aged 35 years or older. In this stratum, the prevalence of physical inactivity was 21.3% among women and 16.7% among men.

We used adjusted RRs from a meta-analysis (18) for the 5 selected diseases, stratified by level of physical activity. Specifically, we compared physical inactivity with both a low level of physical activity and a high level of physical activity to conduct a sensitivity analysis (Table 1).

**Table 1:** Relative Risk (RR) Estimates From Meta-Analysis of Physical Activity^a^, by Disease and Level of Physical Activity

Level of activity^b^	Breast cancer	Colorectal cancer	Diabetes type 2	Ischemic heart disease	Ischemic stroke
Low					
RR	0.967	0.903	0.857	0.837	0.843
1/RR	1.034	1.107	1.166	1.195	1.186
High					
RR	0.863	0.789	0.722	0.754	0.736
1/RR	1.158	1.267	1.385	1.326	1.358

a Source: Kyu et al (18). To assess the impact of physical inactivity, we inverted these RRs, thereby obtaining the RRs associated with physical inactivity compared with the reference levels of high and low physical activity.

b Low was defined as 600–3,999 metabolic equivalent minutes (MET-min/wk) of total activity per week, and high was defined as ≥8,000 MET-min/wk.

### Prevalence and incidence of health consequences

Incidence rates were standardized to the Moroccan population. Among women, the standardized incidence of breast cancer was 49.3 per 100,000. For colorectal cancer, the standardized incidence was 10.0 per 100,000 in women and 10.8 per 100,000 in men. The prevalence of type 2 diabetes was 15.6% in women and 10.8% in men. For ischemic heart disease, the prevalence was estimated at 2.0% in both sexes, while ischemic stroke had a prevalence of 0.69% in both sexes.

### Modeling results

For breast cancer in women, the attributable fraction linked to physical inactivity was 0.88% for a low level of physical activity and 3.95% for a high level of physical activity. For colorectal cancer, the fractions were 2.72% (low) and 6.49% (high) in women, and 1.69% (low) and 4.13% (high) in men. For type 2 diabetes, the attributable fraction increased from 4.16% to 9.10% in women and from 2.60% to 5.84% in men. For ischemic heart disease, the fractions rose from 3.99% to 6.49% in women and from 3.15% to 5.16% in men. For ischemic stroke, the attributable fractions were 3.81% (low) and 7.08% (high) in women, and 3.01% (low) and 5.64% (high) in men.

### Cases attributable to physical inactivity

Table 2 presents the number of disease cases attributable to physical inactivity, stratified by sex and level of physical activity. These values represent the preventable cases of disease if physical inactivity were eliminated. For breast cancer in women, 49 new cases were attributable to a low level of physical activity and 222 to a high level. For colorectal cancer, 31 new cases in women and 20 in men were attributable to a low level of physical activity, whereas 74 in women and 49 in men were attributable to a high level. For type 2 diabetes, 73,930 cases in women and 30,937 in men were linked to a low level of physical activity, and 161,801 in women and 69,456 in men to a high level. For ischemic heart disease in adults aged 35 years or older, 2,670 cases in women and 7,019 in men were attributable to a low level of physical activity, and 4,343 in women and 11,497 in men to a high level. For ischemic stroke in adults aged 35 years or older, 1,147 cases in women and 1,017 in men were attributable to a low level of physical activity, and 2,135 in women and 1,905 in men to a high level (Table 2).

**Table 2:** Number of Cases Attributable to Physical Inactivity, by Sex and Activity Level, Morocco, 2017

Disease	Level of activity^a^	Male		Female		Total no. cases attributable to physical inactivity
		Total cases	No. cases attributable to physical inactivity	Total cases	No. cases attributable to physical inactivity	
Breast cancer	Low	—	—	5,619	49	—
	High				222	
Colorectal cancer	Low	1,190	20	1,140	31	51
	High		49		74	123
Diabetes type 2	Low	1,189,857	30,937	1,778,026	73,930	104,867
	High		69,456		161,801	231,257
Ischemic heart disease (age ≥35 y)	Low	222,810	7,019	66,910	2,670	9,689
	High		11,497		4,343	15,840
Ischemic stroke (≥35 y)	Low	33,769	1,017	30,151	1,147	2,164
	High		1,905		2,135	4,040

Abbreviation: —, not applicable.

a Low was defined as 600–3,999 MET-min/wk, and high was defined as ≥8,000 MET-min/wk.

### Sensitivity analysis

**Figure 1:**
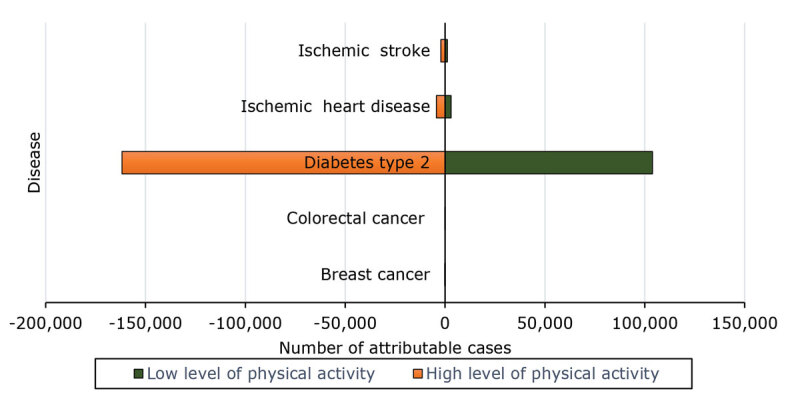
Tornado diagram showing the results of the univariate sensitivity analysis in women of attributable cases of disease by difference in physical activity levels, Morocco, 2017. A low level of physical activity was defined as 600 to 3,999 MET-min/wk and a high level of physical activity as ≥8,000 MET-min/wk. Numbers for colorectal cancer and breast cancer are too small to display on this chart.

**Figure 2:**
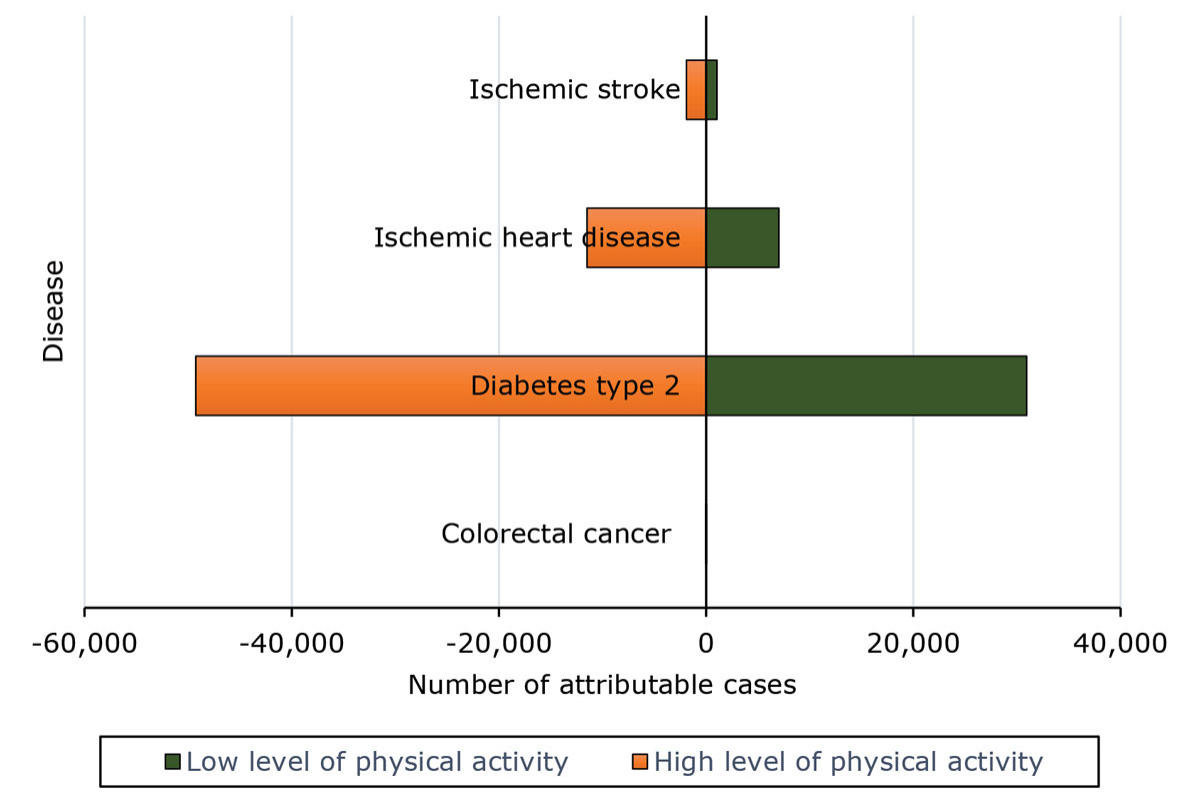
Tornado diagram showing the results of the univariate sensitivity analysis in men of attributable cases of disease by difference in physical activity levels, Morocco, 2017. A low level of physical activity was defined as 600 to 3,999 MET-min/wk and a high level of physical activity as ≥8,000 MET-min/wk. Numbers for colorectal cancer are too small to display on this chart.

For ischemic heart disease and ischemic stroke, there was a substantial reduction in the number of attributable cases when the level of physical activity changed from a low level to a high level in both men and women (Figure 1 and Figure 2). This reduction was more pronounced in men for ischemic heart disease, while the decrease in ischemic stroke cases is notable in both sexes.

**Table:** 

Disease	Low level of physical activity	High level of physical activity
Breast cancer	49	−222
Colorectal cancer	31	−74
Diabetes type 2	73,930	−161,801
Ischemic heart disease	2,670	−4,343
Ischemic stroke	1,147	−2,135

**Table:** 

Disease	Low level of physical activity	High level of physical activity
Colorectal cancer	20	−49
Diabetes type 2	30,937	−69,456
Ischemic heart disease	7,019	−11,497
Ischemic stroke	1,017	−1,905

For type 2 diabetes, the number of attributable cases decreased substantially with higher levels of physical activity, with a greater reduction observed in women compared with men.

For breast cancer, the number of attributable cases decreased in women with increased physical activity. For colorectal cancer, both men and women benefit from a higher level of physical activity, with a reduction in attributable cases.

## Discussion

To our knowledge, this is the first study to estimate the prevalence of NCDs associated with physical inactivity and to examine differences according to sex and level of physical activity in Morocco. The results demonstrate that physical inactivity contributes substantially to the burden of several major diseases. For breast cancer, the attributable fraction ranged from 0.88% for a low level of physical activity to 3.95% for a high level in women. Similarly, for colorectal cancer, up to 6.49% of cases in women and 4.13% in men could be prevented through adequate physical activity. These proportions are lower than the global estimate of 10.4% (5.7%–13.8%) reported in a study using adjusted RRs (4). For type 2 diabetes, the attributable fractions indicate a considerable burden associated with physical inactivity. In women, the attributable fraction ranged from 4.16% for a low level of activity to 9.10% for a high level, whereas in men, the corresponding fractions were 2.60% and 5.84%. These findings align with those of Lee et al, who reported a PAF of 7.2% (3.9%–9.6%) for both sexes combined (4). For ischemic heart disease in adults aged 35 years or older, the attributable fraction ranged from 3.99% to 6.49% in women and from 3.15% to 5.16% in men. These results are consistent with the 5.9% (4.2%–7.6%) reported in Central Asia, the Middle East, and North Africa (26). For ischemic stroke, up to 7.08% of cases in women and 5.64% in men could be prevented by maintaining a high level of physical activity, which is in line with the 5.9% (3.5%–8.5%) reported in North Africa (26).

These findings indicate that increased physical activity is associated with a significant reduction in cases of these diseases among both sexes, but with notable variations. Women particularly benefit from increased physical activity for preventing type 2 diabetes, whereas men see a greater reduction in ischemic heart disease. These differences suggest that physical activity promotion strategies need to be adapted to maximize their effectiveness by considering sex variations and encouraging higher levels of physical activity for better prevention of chronic diseases. These results also suggest that adopting an active lifestyle can play a crucial role in preventing these types of cancer.

### Strengths and limitations

The strengths of our study lie in its comprehensive coverage of major NCDs. This broad scope provides a holistic understanding of the role of physical activity in preventing these health conditions. By stratifying the results by sex, our study highlights important differences between men and women, offering valuable insights for developing targeted and effective public health recommendations. For instance, the greater prevalence of type 2 diabetes in women and ischemic heart disease in men underscores the need for sex-specific interventions.

The use of attributable fractions to quantify the effects of physical inactivity is another key strength, as it provided precise estimates of the number of preventable cases. This approach offered tangible evidence of the benefits of physical activity and strengthened the case for its promotion as a public health priority. The synthesis of existing literature and the integration of robust methodologic approaches enhanced the reliability and generalizability of our findings. These elements contributed to the robustness of our conclusions and provided a solid foundation for future research and policymaking.

Our study has several limitations. First, the results primarily concerned adults aged 18 years or older, except for ischemic heart disease and ischemic stroke, for which the data focused on adults aged 35 or older. This age-specific restriction highlighted a significant limitation: the lack of more detailed data stratified by age group. As a result, the findings reflect overall estimates based on aggregated data rather than a detailed analysis by age, which may limit their precision and applicability to specific age cohorts. Another important limitation is the absence of certain essential national data. This gap required the use of international references to complete our analysis. Although these international sources are reliable and widely recognized, they may not fully capture the specifics of the Moroccan context, such as cultural, socioeconomic, or lifestyle factors. This could affect the accuracy and relevance of the conclusions drawn, particularly for generalizability to the Moroccan population.

### Implications for public health practice, research, and education

Morocco has demonstrated its commitment to promoting physical activity through the National Multisectoral Strategy for the Prevention and Control of Noncommunicable Diseases (2019–2029) (27). However, this study highlights the need to reinforce these efforts with data-driven recommendations. Our results provide a solid foundation for developing new guidelines and improving existing programs, thereby enhancing the prevention of NCDs.

Despite its limitations, this study offers valuable insights into public health policy, research, and education. For public health, our findings can guide the calculation of the economic cost of physical inactivity, facilitate cost-effectiveness evaluations of interventions, and help estimate the economic burden associated with inactivity. These insights can guide the design of more effective and targeted physical activity promotion programs, particularly those tailored to sex-specific needs.

For research, our results provide a robust basis for future studies, including the development of new evaluation indicators and predictive models to assess future trends in physical inactivity and its health and economic consequences. This could pave the way for more nuanced and context-specific analyses.

In the field of education, these findings enrich pedagogical resources by illustrating the importance of physical activity and the costs associated with inactivity. They can be used to raise awareness among students and health professionals, preparing them to design, implement, and evaluate health promotion programs that address the specific needs of diverse populations.

### Conclusion

Promoting physical activity should be a priority for policymakers and health professionals seeking to reduce the burden of NCDs and improve population health. Our study underscores the importance of physical activity in achieving these goals and provides a basis for estimating the economic burden of physical inactivity, evaluating intervention effectiveness, and developing targeted programs. It also offers a foundation for future research, including the development of new evaluation indicators, and for education by emphasizing the health and economic costs of inactivity. Targeted interventions across sex and age groups are essential to maximizing the health benefits of physical activity.
